# Systematic Review with Network Meta-Analysis: Comparative Efficacy and Safety of Combination Therapy with Angiotensin II Receptor Blockers and Amlodipine in Asian Hypertensive Patients

**DOI:** 10.1155/2019/9516279

**Published:** 2019-11-11

**Authors:** Dae Wook Lee, Mira Jung, Hye Won Wang, Zainah Khan, Philippe Pinton

**Affiliations:** ^1^Takeda Pharmaceutical Ltd., Medical Affairs, Seoul, Republic of Korea; ^2^Health Economics and Outcomes Research, Real World Insights, IQVIA, Singapore; ^3^Takeda Pharmaceutical Ltd., Medical Affairs, Japan Pharma, Tokyo, Japan

## Abstract

**Background:**

Hypertension (HTN) is the leading risk factor for cardiovascular mortality globally. The WHO estimates a 60% increase in Asian HTN patients between 2000 and 2025. Numerous studies have compared safety and efficacy between antihypertensive classes, but in-class comparisons of angiotensin II receptor blockers (ARBs) in combination therapy (CT) (fixed-dose combination or dual combination) with a calcium channel blocker (CCB) are lacking in Asia.

**Objective:**

To compare the efficacy and safety of the various ARB-amlodipine CTs and amlodipine (AML) monotherapy for treatment of HTN in Asian population.

**Methods:**

A systematic literature review sourced Asian randomized controlled trials (RCTs) from PubMed and Cochrane Libraries to inform a network meta-analysis (NMA). We considered the ARB-AML CT. The primary efficacy and safety endpoints were short-term (8–12 weeks) treatment response and treatment-emergent adverse events (TEAEs), respectively. AML monotherapy was used as a comparator to allow for indirect treatment effect estimation in the absence of direct RCTs evidence comparing the different ARB-AML CTs.

**Results:**

The analysis included 1198 Asian HTN patients from seven studies involving six ARB-AML CTs: azilsartan (AZL), candesartan (CAN), fimasartan (FIM), losartan (LOS), olmesartan (OLM), and telmisartan (TEL). Compared to AML monotherapy, CT of AZL-AML had five times greater odds of prompting a treatment response (OR 5.2, 95% CI: 2.5, 11.2), while CAN-AML had 3.9 (95% CI: 2.5, 6.4), FIM-AML had 3.4 (95% CI: 1.4, 8.5), TEL-AML had 3.3 (95% CI: 1.6, 7.1), OLM-AML had 2.7 (95% CI: 1.6, 5.0), and LOS-AML had 2.0 (95% CI: 0.6, 7.3). All ARB-AML CTs had safety profiles comparable to AML monotherapy except TEL-AML, which had significantly lower odds of TEAEs (0.26 (95% CI: 0.087, 0.70)).

**Conclusion:**

This study suggests that all ARB-AML CTs compared favorably to AML monotherapy regarding short-term treatment response in uncomplicated HTN patients of Asian origin. AZL-AML prompted the most favorable treatment response. Safety profiles among the ARB-AML CTs were largely comparable. Due to the limited study size and small number of trials (direct evidence), our findings should best be interpreted as an exploratory effort importance to inform future research direction.

## 1. Introduction

Hypertension (HTN) is on the rise globally. The World Health Organization estimated a 60% increase in HTN diagnoses between 2000 and 2025. With 200 million HTN patients in China alone, East Asia is predicted to contribute a third of the projected growth due to fast urbanization and gradual westernization of diet [1, 2]. Korea has the highest prevalence of HTN in Asia, with 67% of elderly presenting with the diagnosis [3]. HTN is considered the most prevalent risk factor for cardiovascular disease (CVD) [4], and the risk for developing HTN increases with age [5]. Antihypertensives help to fill the gaps of blood pressure (BP) control after lifestyle changes. Their utilization has grown rapidly in Asia, doubling between 2007 and 2012 in China alone [6]. Available antihypertensives in Asia include the renin-angiotensin-aldosterone system (RAAS) inhibitors such as angiotensin-converting enzyme inhibitors (ACEIs), angiotensin II receptor blockers (ARBs), *β*-blockers, calcium channel blockers (CCBs), and diuretics.

International and Asian HTN guidelines recommend CT if monotherapy proves ineffective. Combination of CCBs and RAAS inhibitors represents one of the commonly used CTs due to its demonstrated efficacy and favorable safety profile [7, 8]. RAAS inhibitors have shown distinct cardio- and renoprotective advantages [8–11]. ARBs are better tolerated than ACEIs, which are typically associated with dry cough and angioedema [12–15]. This preference is reflected in the rapid growth in the use of CCBs and ARBs that are the most commonly prescribed agents in both Japan and China [1, 6]. Currently, more than 60% of the Japanese HTN patients are treated by a CCB, an ARB, or both [16]. The pleiotropic effects of combining a CCB and ARB include enhancement of nitric oxide bioavailability, anti-inflammatory activity, and inhibition of oxidative stress. The two agents together create a multiplicity of mechanisms available to tackle endothelial dysfunction, reducing the necessary dosage for both agents to trigger adequate response and, thus, reducing side effects [17, 18]. Amlodipine (AML) is a long-acting CCB, which has a longer half-life than second-generation CCB agents and a slow onset, features widely held to be associated with a reduction in reflex sympathetic stimulation [19]. The safety and efficacy of AML therapy has been well established [20] and is, therefore, the most widely used CCB in the Asian region.

A 2015 meta-analysis focusing on global ARB literature has discussed the superiority of the combination of ARB and AML relative to other antihypertensive CTs [11]. But this study did not include CT of some ARBs currently in the market, such as candesartan, and latest in class, fimasartan and azilsartan. Candesartan and azilsartan have unique molecular structures [21], making them particularly important comparators in determining the maximal capacity in-class [12, 21–25] and across antihypertensives [26]. More broadly, there is a paucity of head-to-head randomized controlled trials (RCTs) or comparative analyses assessing between the most up-to-date antihypertensive therapies currently in the market, especially in the Asian region. This study aimed to compare directly and indirectly the efficacy and safety among the ARB-AML CTs for treatment of hypertension in Asian population through a network meta-analysis. The indirect comparison utilizes AML monotherapy as the common comparators.

## 2. Methods

### 2.1. Search Strategy and Study Selection

We searched PubMed and the Cochrane Library from inception to May 2018. We included only East and Southeast Asian RCTs that had at least one arm featuring the CTs of any ARB and AML and another arm featuring AML monotherapy or placebo. Studies without one of these common comparators were excluded through a screening process. Details of the search strategies are described in [Supplementary-material supplementary-material-1].

### 2.2. Type of Interventions

ARB-AML CT included in the literature search were azilsartan (AZL), candesartan (CAN), fimasartan (FIM), irbesartan (IRB), losartan (LOS), olmesartan (OLM), telmisartan (TEL), and valsartan (VAL). Combination with AZL, LOS, OLM, and TEM was fixed-dose combinations. Others were dual combinations. Amlodipine monotherapy (AML) and placebo (PBO) were the common comparator arms. S-amlodipine is an enantiomer of amlodipine, and 2.5 mg of S-amlodipine is equivalent to 5 mg of amlodipine [27]. AZL refers either to azilsartan or azilsartan-medoxomil.

### 2.3. Outcomes of Interest

The primary efficacy and safety outcomes of interest were short-term (8–12 weeks) treatment response and treatment-emergent adverse event (TEAEs), respectively. A patient was considered to have achieved a treatment response if they (1) experienced a reduction in diastolic blood pressure (DBP) ≥ 10 mmHg and/or in systolic blood pressure (SBP) ≥ 20 mmHg and or (2) achieved DBP of <90 mmHg or SBP of <140 mmHg at 8–12 weeks. A TEAE was defined as any undesirable event occurred or worsened in intensity or frequency following the subject's enrolment into the considered studies. For all the considered studies, enrolment was defined after the first dose of the combination therapy. Secondary outcomes were absolute changes of systolic and diastolic BP (in mmHg) from baseline (study enrolment or randomization) to 8–12 weeks postintervention.

### 2.4. Data Extraction and Quality Assessment

Two reviewers independently screened the titles and abstracts of the retrieved citations to identify potentially relevant studies. Relevant data were extracted using a standardized extraction form. Extracted data dimensions include study characteristics, patient characteristics, interventions, outcomes, and study designs. Two other reviewers cross-checked the extracted data randomly and any discrepancies were resolved through consensus. The Cochrane Collaboration's risk of bias assessment tool was used to assess the study-level risk of bias [28].

### 2.5. Data Synthesis and Statistical Analysis

The relative effects of interventions in terms of odds ratio (OR), with 95% confidence interval (CI), were estimated based on data reported by the individual studies. A direct meta-analysis was applied for pooling of the effect estimates using a fixed-effects model if more than one effect estimates were available per comparison. Statistical heterogeneity was assessed using the I-squared statistic if there were sufficient studies available for each pairwise comparison.

A model of network meta-analysis was applied to the available data for the estimation of the direct and indirect effect per pairwise comparison. A Bayesian fixed-effect model with consistency assumption was used [29]. The GeMTC package [30] of *R* was used to implement the network meta-analysis. The GeMTC package is an interface to the JAGS algorithm that executes the Bayesian estimation of the model parameters through a Markov chain Monte Carlo (MCMC) process. Default priors for treatment effect and heterogeneity parameters were used in all analyses.

Rank analysis was also conducted. Rank analysis refers to the estimation of the probabilities that indicate how likely each treatment options may be the best, second best, and so on, among the comparators in the analysis. The treatments were ranked by their effects relative to a baseline when the MCMC process was implemented. A frequency table was constructed from the rankings and normalized by the number of iterations to give the rank probabilities. To rank the intervention hierarchy in the network meta-analysis, the surface under the cumulative ranking (SUCRA) curves and the mean ranks were estimated [31]. The rankings for safety and efficacy were then combined and summarized in a clustered ranking plot.

Publication bias was not examined due to the limited number of available studies per comparison. This study protocol is reported according to the Preferred Reporting Items for Systematic Reviews and Meta-Analyses (PRISMA) extension statement for network meta-analysis [32]. All analyses were performed in *R* statistical programming version 3.4.4. A two-sided *p*-value of ≤ 0·05 was considered statistically significant.

## 3. Results

### 3.1. Study Selection and Risk of Bias

**Figure 1:**
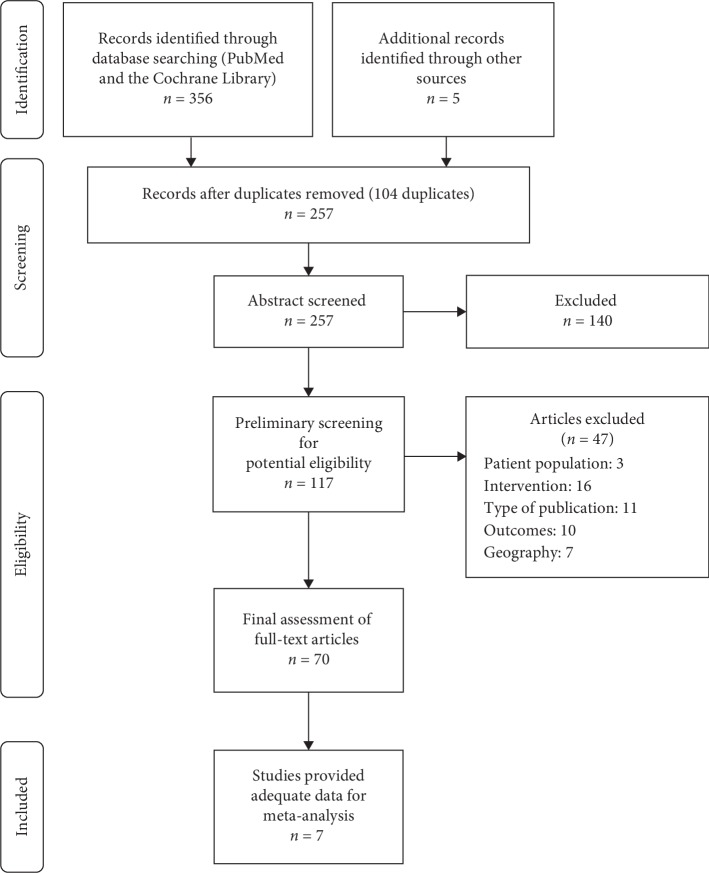
PRISMA diagram for systematic literature review.

We identified 257 records, in which 117 potentially eligible articles were preliminary screened in full text. This preliminary screening process excluded 47 papers. A final, full-text assessment was then performed for the remaining 70 papers. Of these 70 papers, seven studies fulfilled the study eligibility criteria and provided adequate data for the intended analysis. The remaining 110 articles were excluded due to the absence of the interested study outcomes, being nonrandomized studies, being nonprimary (post hoc) analysis, and being duplicated publication.  Figure 1 presents the PRISMA flow diagram that illustrates the study selection processes. We did not find any study with adequate data on VAL-AML and IRB-AML CTs that are suitable for inclusion in this network meta-analysis.

Most studies contained evidence of low risk of bias. Most uncertainties arise from insufficient reporting of the study blinding methods ([Supplementary-material supplementary-material-1]). Incomplete outcome data, selective outcome reporting, and other biases were the domains that were found to pose high risk of bias in at least two out of the six included studies. None of them showed evidence of definite high risk of bias in terms of random sequence generation, allocation concealment, blinding of participants, and outcome assessment.

### 3.2. Characteristics of Included Studies and Network

Five out of the seven studies [33–39] were published within the recent five years (2014–2018). These studies were conducted in Japan, Korea, China, and Taiwan. The study-level characteristics are available in [Supplementary-material supplementary-material-1]. We did not find any literature from the Southeast Asia region. All trials evaluated the use of ARB-AML CT and AML as monotherapy. The average age of participants was 55.8 years (range 52.4–58.9 years). The average proportion of male participants was 66.8% (range 49.7%–86.0%). Characteristics of the participants for each study are summarized in [Supplementary-material supplementary-material-1].

Network diagrams of all the eligible comparisons for primary and secondary efficacy outcomes are presented in part figures (a) and (b) in [Supplementary-material supplementary-material-1]. Treatments included in the efficacy outcome network diagram are CT of fimasartan-AML (FIM-AML), losartan-AML (LOS-AML), olmesartan-AML (OLM-AML), telmisartan-AML (TEL-AML), azilsartan-AML (AZL-AML), and candesartan-AML (CAN-AML), along with AML and PBO. Network structure of all the eligible comparison for the safety outcome is similarly presented in part figure (b) in Appendix. The network structure for safety outcome comparison was largely similar except with the absence of the OLM-AML CT. No eligible study was found to have provided adequate safety data on OLM-AML CT suitable for our analysis.

### 3.3. Primary Efficacy and Safety Outcomes

**Figure 2:**
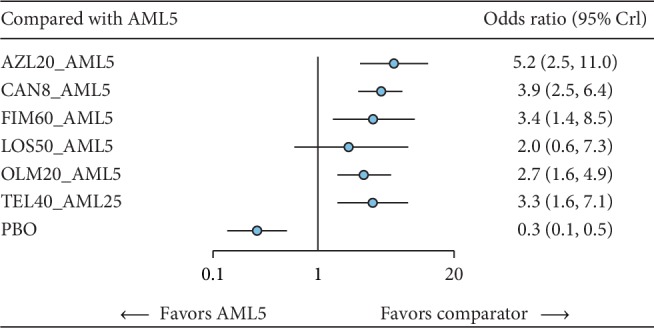
Direct treatment effect on response relative to AML monotherapy. AML, amlodipine; AZL, azilsartan; CAN, candesartan; FIM, fimasartan; OLM, olmesartan; TEL, telmisartan; LOS, losartan; PBO, placebo; CI, confidence interval.

Direct treatment effects comparing each available CT against the AML monotherapy for primary efficacy and safety outcome are synthesized and presented in Figure 2.

#### 3.3.1. Treatment Response

Seven studies involving 1198 participants have provided suitable data for the evaluation of short-term treatment response among the six different ARB-AML CTs via a network meta-analysis. We found that all except LOS-AML CT have shown a statistically significant increase in the odds of achieving a treatment response in comparison with AML monotherapy (Figure 2). Although confidence intervals overlapped among the ARB-AML CTs, AZL-AML CT had the greatest odd of prompting a treatment response compared to AML monotherapy (OR 5.2, 95% CI: 2.6, 11.0) in terms of midpoint estimate. This was followed by CAN-AML (OR 3.9, 95% CI: 2.5, 6.4), FIM-AML (OR 3.4, 95% CI: 1.4, 8.4), TEL-AML (OR 3.3, 95% CI: 1.6, 7.0), OLM-AML (OR 2.8, 95% CI: 1.6, 4.9), and LOS-AML (OR 2.0, 95% CI: 0.6, 7.3).  Table 1 shows a summary matrix of all direct and indirect comparisons among the included comparators.

#### 3.3.2. Treatment-Emergent Adverse Events

**Figure 3:**
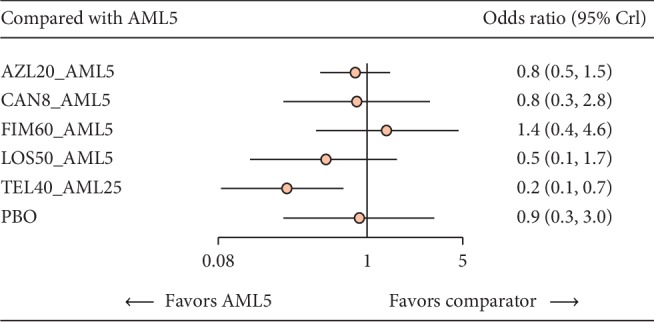
Direct treatment effect on TEAEs relative to AML monotherapy. AML, amlodipine; AZL, azilsartan; CAN, candesartan; FIM, fimasartan; TEL, telmisartan; LOS, losartan; PBO, placebo; CI, confidence interval.

Six studies involving 678 participants provided suitable data for the evaluation of short-term TEAEs. Five different ARB-AML CTs were compared via a network meta-analysis. Data on OLM-AML CT were not available. We found that all except TEL-AML CT had largely comparable results on TEAEs in comparison with AML monotherapy (Figure 3). TEL-AML CT showed lower odds of experiencing a TEAE when compared to AML monotherapy (OR 0.26, CI: 0.09–0.70). Direct and indirect effect estimates comparing the TEAE rate of different ARB-AML CTs are shown in Table 2.

**Figure 4:**
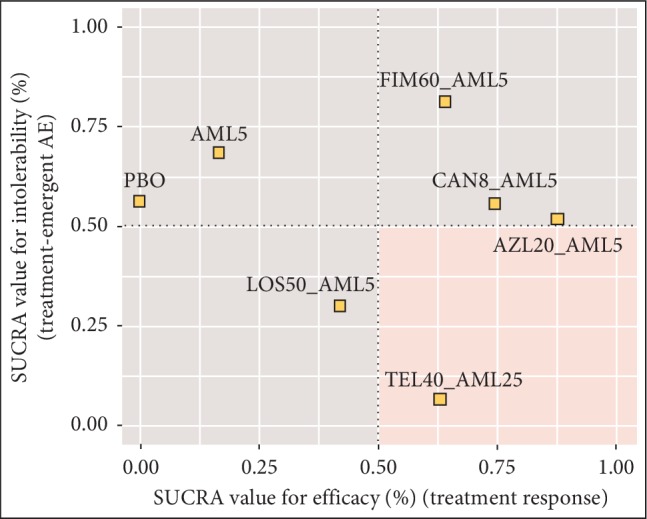
Clustered ranking plot of SUCRA values for efficacy vs. intolerability. AML, amlodipine; AZL, azilsartan; CAN, candesartan; FIM, fimasartan; LOS, losartan; TEL, telmisartan; PBO, placebo; S-AML, active enantiomer of amlodipine; CI, confidence interval.

The clustered ranking plot in Figure 4 demonstrated that AZL-AML CT is associated with the highest probability of achieving a short-term treatment response (SUCRA value 87.5%) while maintaining a comparable tolerability profile (in terms of TEAEs, SUCRA value 51.8%) to other ARB-AML CTs. FIM-AML appears to carry a higher risk of TEAEs. [Supplementary-material supplementary-material-1] illustrates the SUCRA curves of each comparator for treatment response and TEAE comparison.

### 3.4. Secondary Efficacy Outcomes

#### 3.4.1. Blood Pressure Change from Baseline

The estimated mean differences of systolic and diastolic BP changes from baseline comparing the ARB-AML CTs to AML monotherapy are presented in [Supplementary-material supplementary-material-1]. The absolute effect magnitudes appear to follow a pattern similar to that observed in the analysis of the primary efficacy outcome. Indirect effect estimates compared among the CTs for mean differences of systolic and diastolic BP changes are shown in [Supplementary-material supplementary-material-1].

## 4. Discussion

### 4.1. Statement of Principle Findings

This network meta-analysis compared six ARB-AML CTs from seven RCTs involving 1198 patients with uncomplicated HTN. Overall, the expected class effects of ARB-AML CT in both efficacy and safety are observed with distinct hierarchy among them. Based on the indirect comparison, AZL-AML CT appears to have the greatest probability for being the most potent agent in achieving a treatment response in 8–12 weeks followed by CAN-AML, FIM-AML, and TEL-AML. Secondary efficacy endpoints of 8–12 weeks mean change in DBP and SBP (from baseline) show a similar ranking pattern. All direct comparisons with AML monotherapy in efficacy endpoints were statistically significant.

All CTs had safety profile close to that of AML monotherapy, except TEL-AML, which showed a higher likelihood of having lower TEAE than other ARB-AML CTs. This outlying safety feature of TEL-AML may be explained by the variation of its compound structure featured in its combination. That is, all studies except for the one regarding TEL-AML featured amlodipine 5 mg, while the study concerning TEL-AML featured the enantiomer S-amlodipine at 10 mg [40]. Literature has previously described these two molecules at their respective strengths to be equivalent in their efficacy; however, this determination was made based on monotherapy [27]. Due to the limited study size and small number of trials (direct evidence), our findings here should best be interpreted as an exploratory effort importance to inform future research direction.

### 4.2. Relation of Findings to Previous Research

Parallel to our findings, a recent comparative ARB study noted the distinct efficacious advantage of AZL in monotherapy relative to other ARBs. Other qualitative studies also noted AZL monotherapy's greater effect on treatment response and better reductions in BP compared to other ARB monotherapies such as valsartan, olmesartan, and candesartan [24, 41]. This superiority may be potentially linked to the unique molecular structure of AZL. AZL contains an oxo-oxadiazole ring, which allows for higher persistent of inhibitory action on the angiotensin II type 1 (AT_1_) receptor [21, 25] compared to other similar ARBs. It is currently unsure, yet if the addition of AML to AZL has a synergistic effect on BP outcomes. In addition, ARBs are known for their added benefit on end-organ protection. Telmisartan particularly has been suggested to be the preferred choice for ARB-based CT for patients with concomitant metabolic disorders and evidence of renal disease for its unique characteristics. Unfortunately, the limited number available patients considered in this analysis did not allow the evaluation of these added benefits. Our findings challenged such preference and reinforced the need of head-to head clinical trials in order to inform the right clinical decision of antihypertensive use, particularly when deciding which CT should be used.

### 4.3. Strengths and Limitations

This study represents an advancement in the global and Asian comparative antihypertensive literature due to the limited availability of comparative studies focusing on quantitative, in-class comparisons of high-potency combinations of preferred antihypertensives to date. The study supplements a recent meta-analysis on ARB-AML CTs relative to AML monotherapy [11] by including a wider variety of ARBs to date (e.g. fimasartan and azilsartan). Other assessments in the past have typically featured qualitative comparisons with broad scope of analyses: one comparing between anti-hypertensive classes [42], a few exploring multiclass combinations [43, 44], and others comparing ARB monotherapies in a variety of subpopulations [45–47].

A major limitation of the study was the small collection of trials being compared. According to the best practices in indirect analyses, this study features only half the number of trials and approximately one quarter of the required study population size per comparison to achieve the precision of a direct analysis [48]. Hence, findings from this analysis should best be seen as an exploratory effort and be interpreted within the limitation of small study size and limited power. Another limitation was the variability in response rate definitions among the RCTs featured in this comparison, and most conflated the concept with BP control. While the study was intended for all parts of Asia, literature for robust comparison was available only from East Asia, notably China, Japan, and South Korea, and thus, results may not be generalizable to other study populations. The comparison was based only on short-term endpoint analysis and only one type of combination was considered. Further, paucity in clinical outcomes data limits this comparative study in making long-term clinical implications. Last, the aggregated quality standards of the included studies, though, did show overall low risk of bias (53%), particularly in methods of randomization and blinding of interventions from participants and personnel. There were moderate levels of uncertainty in the risk associated with blinding of outcomes and allocation concealment (29%) and some high risk of other forms of bias such as extrapolated imputations, directional bias-like industry sponsorship, attrition due to AEs, and lack of efficacy (18%).

## 5. Conclusion

This Asian-focused analysis suggests the possibility that AZL-AML CT might be able to offer a marginally greater short-term treatment response for uncomplicated hypertension relative to other ARB-AML CTs. All ARB-AML CTs in comparison showed safety profiles comparable to AML monotherapy and placebo. To our knowledge, this is likely the first comparative study of efficacy and safety that has considered the latest range of ARB-AML CT in Asia. Head-to-head RCTs might be helpful to confirm these suggestive findings. Long-term studies might be of great value to evaluate the long-term clinical effects. Due to the limited study size and small number of trial (direct evidence), our findings should best be interpreted as an exploratory effort importance to inform future research direction.

## Figures and Tables

**Table 1:** Summary matrix showing all pairwise comparisons for treatment response.

*AML5*	**5.21** (2.53, 11.19)	**3.93** (2.46, 6.43)	**3.37** (1.41, 8.47)	**1.97** (0.6, 7.3)	**2.74** (1.57, 4.94)	**3.29** (1.58, 7.09)	**0.26** (0.13, 0.49)
0.19 (0.09, 0.39)	*AZL20_AML5*	0.75 (0.31, 1.81)	0.65 (0.2, 2.08)	0.38 (0.09, 1.68)	0.53 (0.2, 1.33)	0.63 (0.22, 1.8)	0.05 (0.02, 0.13)
0.25 (0.16, 0.41)	1.33 (0.55, 3.27)	*CAN8_AML5*	0.86 (0.32, 2.36)	0.5 (0.14, 2.02)	0.7 (0.33, 1.48)	0.84 (0.34, 2.06)	0.07 (0.03, 0.14)
0.3 (0.12, 0.71)	1.55 (0.48, 4.97)	1.17 (0.42, 3.08)	*FIM60_AML5*	0.59 (0.13, 2.84)	0.82 (0.28, 2.32)	0.98 (0.3, 3.12)	0.08 (0.03, 0.19)
0.51 (0.14, 1.66)	2.64 (0.6, 11.01)	1.99 (0.5, 7.19)	1.7 (0.35, 7.69)	*LOS50_AML5*	1.39 (0.33, 5.25)	1.66 (0.37, 6.93)	0.13 (0.03, 0.51)
0.36 (0.2, 0.64)	1.9 (0.75, 4.9)	1.43 (0.68, 3.02)	1.22 (0.43, 3.56)	0.72 (0.19, 2.99)	*OLM20_AML5*	1.2 (0.47, 3.14)	0.09 (0.04, 0.22)
0.3 (0.14, 0.63)	1.59 (0.56, 4.54)	1.19 (0.49, 2.9)	1.02 (0.32, 3.32)	0.6 (0.14, 2.72)	0.84 (0.32, 2.15)	*TEL40_AML25*	0.08 (0.03, 0.21)
3.82 (2.02, 7.6)	20.1 (7.59, 55.96)	15.04 (7.25, 32.97)	12.91 (5.33, 33.47)	7.61 (1.95, 33.24)	10.54 (4.48, 25.82)	12.63 (4.69, 35.22)	*PBO*

Abbreviations: AML, amlodipine; AZL, azilsartan; CAN, candesartan; FIM, fimasartan; OLM, olmesartan; TEL, telmisartan; LOS, losartan; PBO, placebo; CI, confidence intervals.

**Table 2:** Summary matrix showing all pairwise comparisons for treatment-emergent adverse events.

*AML5*	**0.82** (0.46, 1.47)	**0.84** (0.25, 2.84)	**1.39** (0.43, 4.6)	**0.51** (0.14, 1.65)	**0.26** (0.09, 0.69)	**0.89** (0.25, 3.04)
1.23 (0.68, 2.16)	*AZL20_AML5*	1.03 (0.26, 3.95)	1.7 (0.46, 6.36)	0.62 (0.15, 2.34)	0.32 (0.09, 0.99)	1.08 (0.27, 4.23)
1.19 (0.35, 4.04)	0.97 (0.25, 3.78)	*CAN8_AML5*	1.65 (0.3, 8.99)	0.59 (0.1, 3.28)	0.31 (0.06, 1.49)	1.05 (0.18, 5.95)
0.72 (0.22, 2.31)	0.59 (0.16, 2.16)	0.61 (0.11, 3.29)	*FIM60_AML5*	0.36 (0.06, 1.93)	0.19 (0.04, 0.87)	0.64 (0.19, 2.02)
1.98 (0.6, 7.27)	1.62 (0.43, 6.76)	1.69 (0.3, 9.87)	2.76 (0.52, 16.13)	*LOS50_AML5*	0.51 (0.1, 2.63)	1.76 (0.3, 10.61)
3.84 (1.45, 11.71)	3.15 (1.01, 10.97)	3.28 (0.67, 16.74)	5.38 (1.15, 27.26)	1.95 (0.38, 9.9)	*TEL40_AML25*	3.43 (0.68, 17.85)
1.13 (0.33, 3.99)	0.92 (0.24, 3.71)	0.95 (0.17, 5.49)	1.57 (0.5, 5.27)	0.57 (0.09, 3.29)	0.29 (0.06, 1.46)	*PBO*

Abbreviations: AML, amlodipine; AZL, azilsartan; CAN, candesartan; FIM, fimasartan; TEL, telmisartan; LOS, losartan; PBO, placebo; CI, confidence intervals.

## Data Availability

The data used to support the findings of this study are included within the supplementary information files

## Supplementary Materials

Supplementary Materials Appendix 1: details of search strategies. Appendix 2: details of the risk of bias assessment results. Appendix 3: study-level characteristics of included publication. Appendix 4: characteristics of the study participants. Appendix 5: network diagrams of all the eligible comparisons for primary efficacy and safety outcomes. Appendix 6: SUCRA curves of each comparator for treatment response and TEAE comparison. Appendix 7: direct treatment effect on changes of SBP and DBP from baseline. Appendix 8: direct and indirect treatment effect on changes of SBP and DBP from baseline.
