# Supplementary material for: Systematic Review with Network Meta-Analysis: Comparative Efficacy and Safety of Combination Therapy with Angiotensin II Receptor Blockers and Amlodipine in Asian Hypertensive Patients
Source: Int J Hypertens. 2019 Nov 11;2019:9516279. doi: 10.1155/2019/9516279 (PMC6885253; doi:10.1155/2019/9516279)
Supplement: Supplementary Materials — Appendix 1: details of search strategies. Appendix 2: details of the risk of bias assessment results. Appendix 3: study-level characteristics of included publication. Appendix 4: characteristics of the study participants. Appendix 5: network diagrams of all the eligible comparisons for primary efficacy and safety outcomes. Appendix 6: SUCRA curves of each comparator for treatment response and TEAE comparison. Appendix 7: direct treatment effect on changes of SBP and DBP from baseline. Appendix 8: direct and indirect treatment effect on changes of SBP and DBP from baseline. [file 9516279.f1.docx]

**Supplementary Material**

**Title**: Systematic Review with Network Meta-Analysis: Comparative Efficacy and Safety of Fixed Dose Combinations of Angiotensin II Receptor Blockers and Amlodipine in Asian Hypertension

Contents

[**Appendix 1. Details of search strategies** 2](#_Toc536101974)

[**Appendix 2. Details of the risk of bias assessment results** 3](#_Toc536101975)

[**Appendix 3. Study-level characteristics of included publication** 4](#_Toc536101976)

[**Appendix 4. Characteristics of the study participants** 5](#_Toc536101977)

[**Appendix 5. Network diagrams of all the eligible comparisons for primary efficacy and safety outcomes** 7](#_Toc536101978)

[**Appendix 6. SUCRA curves of each comparator for treatment response and TEAE comparison.** 8](#_Toc536101979)

[**Appendix 7. Direct treatment effect on changes of SBP and DBP from baseline** 9](#_Toc536101980)

[**Appendix 8. Direct and indirect treatment effect on changes of SBP and DBP from baseline** 10](#_Toc536101981)

**Appendix 1. Details of search strategies**

Search strategy for original searches (15 March 2018)

| **No.** | **Search strategy** | **Hits** |
| --- | --- | --- |
| **MEDLINE** | | |
| #1 | Search hypertension[TI] OR hypertensive[TI] OR “blood pressure”[TI] | 2,23,928 |
| #2 | Search ARB[TI] OR “angiotensin receptor blocker”[TI] OR amlodipine[TI] OR CCB[TI] OR “calcium channel blocker”[TI] OR azilsartan[TI] OR olmesartan[TI] OR candesartan[TI] OR eprosartan[TI] OR irbesartan[TI] OR valsartan[TI] OR losartan[TI] OR DuP?753[TI] OR DuP753[TI] OR MK?954[TI] OR Cozaar[TI] OR Cozaarex[TI] OR Lortaan[TI] OR Losaprex[TI] OR “Neo Lotan” [TI] OR Nu-Lotan [TI] OR Oscaar[TI] OR telmisartan[TI] OR fimasartan[TI] OR “fixed dose combination”[TI] OR monotherapy[TI] | 15,998 |
| #3 | Search (Asia[TIAB] OR Asian[TIAB] OR Pacific[TIAB] OR China[TIAB] OR Korea[TIAB] OR Japan[TIAB]) | 3,75,131 |
| #4 | #1 AND #2 AND #3 | 118 |
| **Cochrane** | | |
| #1 | hypertension or hypertensive or "blood pressure”: ti (Word variations have been searched) | 29,321 |
| #2 | ARB or "angiotensin receptor blocker" or amlodipine or CCB or "calcium channel blocker" or azilsartan or olmesartan or candesartan or eprosartan or irbesartan or valsartan or losartan or DuP?753 or DuP753 or MK?954 or Cozaar or Cozaarex or Lortaan or Losaprex or "Neo Lotan" or Nu-Lotan or Oscaar or telmisartan or fimasartan or "fixed dose combination" or monotherapy: ti (Word variations have been searched) | 9,280 |
| #3 | Asia or Asian or Pacific or China or Korea or Japan: ti,ab,kw (Word variations have been searched) | 27,253 |
| #4 | #1 AND #2 AND #3 | 183 |
| **Total initial hits** | | **305** |

Search strategy for updated searches (14 May 2018)

| **No.** | **Search strategy** | **Hits** |
| --- | --- | --- |
| **MEDLINE** | | |
| #1 | Search hypertension[TI] OR hypertensive[TI] OR “blood pressure”[TI] | 2,25,135 |
| #2 | Search ARB[TI] OR “angiotensin receptor blocker”[TI] OR amlodipine[TI] OR CCB[TI] OR “calcium channel blocker”[TI] OR azilsartan[TI] OR olmesartan[TI] OR candesartan[TI] OR eprosartan[TI] OR irbesartan[TI] OR valsartan[TI] OR losartan[TI] OR DuP?753[TI] OR DuP753[TI] OR MK?954[TI] OR Cozaar[TI] OR Cozaarex[TI] OR Lortaan[TI] OR Losaprex[TI] OR “Neo Lotan” [TI] OR Nu-Lotan [TI] OR Oscaar[TI] OR telmisartan[TI] OR fimasartan[TI] OR “fixed dose combination”[TI] OR monotherapy[TI] | 16,137 |
| #3 | Search asia*[TIAB] OR thailand[TIAB] OR thai[TIAB] OR singapore*[TIAB] OR taiwan*[TIAB] OR philippines[TIAB] OR filipino*[TIAB] OR vietnam*[TIAB] OR cambodia*[TIAB] OR laos[TIAB] OR "Hong Kong"[TIAB] OR "indonesia"[TIAB] OR "Malaysia"[TIAB] | 2,41,023 |
| #4 | #1 AND #2 AND #3 | 55 |

**Appendix 2. Details of the risk of bias assessment results**

|  | Selection bias | | Performance bias | Detection Bias | Attrition bias | Reporting bias | Other |
| --- | --- | --- | --- | --- | --- | --- | --- |
|  | *Random sequence generation* | *Allocation concealment* | *Blinding of participants and personnel* | *Blinding of outcome* | *Incomplete outcome data* | *Selective reporting* | *Other sources of bias* |
| Low | 57% | 43% | 100% | 0% | 71% | 71% | 29% |
| Uncertain | 43% | 57% | 0% | 100% | 0% | 0% | 0% |
| High | 0% | 0% | 0% | 0% | 29% | 29% | 71% |

|  | Selection bias | | Performance bias | Detection Bias | Attrition bias | Reporting bias | Other | Industry Sponsored |
| --- | --- | --- | --- | --- | --- | --- | --- | --- |
| *Study* | *Random sequence generation* | *Allocation concealment* | *Blinding of participants* | *Blinding of outcome* | *Incomplete outcome data* | *Selective reporting* | *Other sources of bias* |  |
| 23 | Low | Uncertain | Low | Uncertain | High | Low | High | Y |
| 39 | Low | Low | Low | Uncertain | Low | Low | High | Y |
| 116 | Uncertain | Uncertain | Low | Uncertain | Low | Low | Low | Y |
| 121 | Low | Low | Low | Uncertain | Low | Low | Low | N |
| 122 | Low | Low | Low | Uncertain | Low | Low | Low | Y |
| 237 | Low | Uncertain | Low | Uncertain | Low | High | High | Y |
| 241 | Uncertain | Low | Low | Uncertain | High | High | High | Y |

**Appendix 3. Study-level characteristics of included publication**

| **Sr. no** | **First author (Study Name)** | **Year** | **Trial design** | **Location of trial** | **Study Sample (N)** | **Time point of data capture** | **Inclusion criteria** | |
| --- | --- | --- | --- | --- | --- | --- | --- | --- |
|  |  |  |  |  |  |  | **Patient Population** | **Outcome** |
| 23 | Rakugi *et al* | 2014 | Randomized, Double blind | Japan | 403 | 8-week siSBP/siDBP | Grade 1 to 2 essential hypertension | siSBP, siDBP, Treatment Response, |
| 39 | Rakugi *et al* | 2012 | Randomized, parallel-group, double-blind, placebo-controlled | Japan | 444 | 12-week siBP, RR and target BP levels | Adult patients with mild to moderate essential hypertension | siSBP, siDBP Treatment Response, Target BP |
| 116 | Ihm *et al* | 2016 | Randomized, Double-blind, Parallel group | Korea | 184 | 8-week mean siDBP, siSBP, Response | Adult patients with uncomplicated, mild-to-moderate essential hypertension | Mean siDBP, siSBP, Control rate, Treatment response |
| 121 | Zhu *et al* | 2014 | Randomized, Double blind,  Double-dummy, Active-controlled, Parallel group | China | Study 1: 270  Study 2: 282 | 8-week seSBP, seDBP, Response | Adult patients with mild to moderate essential hypertension | seSBP, seDBP, Treatment response |
| 122 | Park *et al* | 2012 | Randomized, Double-blind, Multicenter | Korea | 320 | 8-week mean siDBP | Adult patients with essential hypertension | siDBP, siSBP, Treatment response |
| 237 | Lee HY *et al* | 2015 | Randomized, Multicenter, Double-blind, Placebo-controlled | Korea | 419 | 8-week siDBP | Hypertensive patients with SiDBP between 90 and 114 mm Hg | siBP, Treatment Response |
| 241 | Sohn *et al* | 2017 | Randomized, Double-blind, Multicenter | Korea | 439 | 8-week siSBP, Response, Control Rate | Adults patients with essential hypertension | siSBP, siDBP, Treatment Response |

**Appendix 4. Characteristics of the study participants**

|  | | | | *Sample Characteristics, mean(SD)/%* | | | |
| --- | --- | --- | --- | --- | --- | --- | --- |
| **ID** | **Study** | **Study Arms** | **Arm Size** | **Age (year)** | **Weight (kg)** | **BMI** | **Male (%)** |
| 39 | Rakugi *2012* | Candesartan/amlodipine 8/5 mg | 101 | 56.6 (10.8) | 65.3 (12.7) | 25.1 (3.6) | 59.4 |
|  |  | Candesartan/amlodipine 8/2.5 mg | 36 | 57.4 (11.5) | 70.8 (16.0) | 26.8 (4.9) | 58.3 |
|  |  | Candesartan/amlodipine 8/0 mg | 100 | 56.9 (11.3) | 70.4 (13.1) | 26.7 (4.1) | 67.0 |
|  |  | Candesartan/amlodipine 0/5 mg | 100 | 56.7 (9.7) | 67.8 (14.0) | 25.7 (4.1) | 64.0 |
|  |  | Candesartan/amlodipine 4/5 mg | 36 | 56.9 (8.3) | 64.6 (12.6) | 25.5 (3.3) | 44.4 |
|  |  | Candesartan/amlodipine 4/2.5 mg | 35 | 55.9 (9.9) | 68.3 (12.6) | 26.3 (3.9) | 54.3 |
|  |  | Placebo | 36 | 58.8 (13.7) | 67.7 (14.4) | 25.7 (3.7) | 69.4 |
| 23 | Rakugi *2014* | Azilsartan/ amlodipine 20/5 mg | 150 | 57.6 (9.8) | 68.9 (13.4) | 25.9 (4.1) | 57.3 |
|  |  | Azilsartan/ amlodipine 0/2.5 mg | 151 | 57.6 (9.8) | 68.3 (13.9) | 25.8 (3.9) | 56.3 |
|  |  | Azilsartan 20 mg | 151 | 58.5 (9.1) | 68.3 (13.9) | 25.9 (4.1) | 64.9 |
|  |  | Amlodipine 5 mg | 75 | 58.9 (9.9) | 67.2 (13.0) | 25.4 (3.7) | 61.3 |
|  |  | Amlodipine 2.5 mg | 76 | 56.4 (11.4) | 70.4 (12.6) | 26.0 (3.8) | 64.5 |
| 116 | Ihm *2016* | S-amlodipine/ telmisartan 2.5/40 mg | 63 | 57.1 (10.5) | 69.2 (10.3) | - | 68.3 |
|  |  | S-amlodipine/ telmisartan 2.5/80 mg | 61 | 58.3 (10.6) | 72.7 (10.6) | - | 72.1 |
|  |  | S-amlodipine 2.5 mg | 60 | 54.9 (8.5) | 73.2 (10.8) | - | 75.0 |
| 121 | Zhu *2014* | Olmesartan medoxomil 40 mg (sub-study 1) | 130 | 53.0 (10.3) | - | 26.4 (3.1) | 59.2 |
|  |  | Olmesartan medoxomil/Amlodipine 20/5 mg (sub-study 1) | 128 | 53.3 (8.9) | - | 26.3 (3.0) | 56.3 |
|  |  | Amlodipine 5 mg (sub-study 2) | 147 | 54.1 (8.7) | - | 26.0 (3.3) | 53.7 |
|  |  | Olmesartan medoxomil/Amlodipine 20/5 mg (sub-study 2) | 142 | 54.1 (9.3) | - | 26.2 (3.0) | 54.9 |
|  |  | Amlodipine 10 mg | 91 | 53.2 (10.3) | 71.2 (10.3) | - | 82.4 |
| 122 | Park 2012 | Amlodipine/Losartan 5/50 mg | 38 | 55.1 (9.2) | 72.0 (12.6) | - | 73.7 |
|  |  | Amlodipine/Losartan 5/100 mg | 41 | 52.5 (8.6) | 70.5 (10.7) | - | 73.2 |
|  |  | Amlodipine/Losartan 10/50 mg | 43 | 50.7 (10.8) | 67.7 (10.5) | - | 62.8 |
|  |  | Amlodipine/Losartan 10/100 mg | 41 | 53.2 (10.6) | 68.5 (12.4) | - | 63.4 |
|  |  | Amlodipine 5 mg | 40 | 53.1 (8.8) | 66.3 (8.9) | - | 67.5 |
|  |  | Amlodipine 10 mg | 39 | 54.8 (10.5) | 69.3 (9.6) | - | 59.0 |
|  |  | Losartan 50 mg | 38 | 50.0 (8.8) | 69.8 (12.1) | - | 68.4 |
|  |  | Losartan 100 mg | 40 | 55.5 (9.6) | 69.8 (10.3) | - | 67.5 |
| 237 | Lee *2015* | Placebo | 50 | 56.5 (8.1) | 69.0 (9.4) | 25.2 (2.5) | 78.0 |
|  |  | AML 5 mg | 44 | 54.7 (8.7) | 71.3 (9.6) | 25.4 (2.8) | 86.0 |
|  |  | AML 5 mg | 44 | 57.8 (9.0) | 71.5 (9.8) | 25.5 (2.8) | 84.0 |
|  |  | FMS 30 mg | 44 | 54.8 (8.7) | 72.8 (11.3) | 26.1 (3.0) | 80.0 |
|  |  | FMS 60 mg | 44 | 56.4 (10.0) | 71.6 (9.5) | 25.5 (2.5) | 80.0 |
|  |  | FMS 30 mg/AML 5 mg | 44 | 54.1 (10.7) | 72.2 (10.4) | 25.5 (3.0) | 84.0 |
|  |  | FMS 30 mg/AML 10 mg | 48 | 54.4 (11.2) | 71.3 (10.7) | 25.9 (3.1) | 71.0 |
|  |  | FMS 60 mg/AML 5 mg | 47 | 54.8 (10.1) | 70.2 (11.5) | 25.5 (3.7) | 70.0 |
|  |  | FMS 60 mg/AML 10 mg | 47 | 54.1 (9.8) | 70.3 (9.7) | 25.0 (2.4) | 79.0 |
| 241 | Sohn *2017* | Candesartan/ Amlodipine 8/5 mg | 53 | 58.9 (11.1) | 70.8 (11.7) | 25.7 (3.4) | 66.0 |
|  |  | Candesartan/ Amlodipine 8/10 mg | 53 | 54.4 (10.4) | 67.8 (9.9) | 24.8 (2.9) | 69.8 |
|  |  | Candesartan/ Amlodipine 16/5 mg | 52 | 55.2 (10.2) | 69.9 (12.7) | 25.5 (3.4) | 75.0 |
|  |  | Candesartan/ Amlodipine 16/10 mg | 54 | 57.4 (12.0) | 69.6 (11.3) | 25.0 (3.0) | 66.7 |
|  |  | Candesartan 8 mg | 51 | 56.8 (10.1) | 70.9 (11.7) | 25.7 (2.9) | 76.5 |
|  |  | Candesartan 16 mg | 56 | 58.9 (9.6) | 70.7 (10.4) | 25.4 (3.0) | 75.0 |
|  |  | Amlodipine 5 mg | 54 | 58.1 (9.4) | 68.2 (10.2) | 25.1 (2.7) | 72.8 |
|  |  | Amlodipine 10 mg | 52 | 58.4 (10.7) | 69.4 (11.1) | 25.0 (3.0) | 73.1 |

**Appendix 5. Network diagrams of all the eligible comparisons for primary efficacy and safety outcomes**


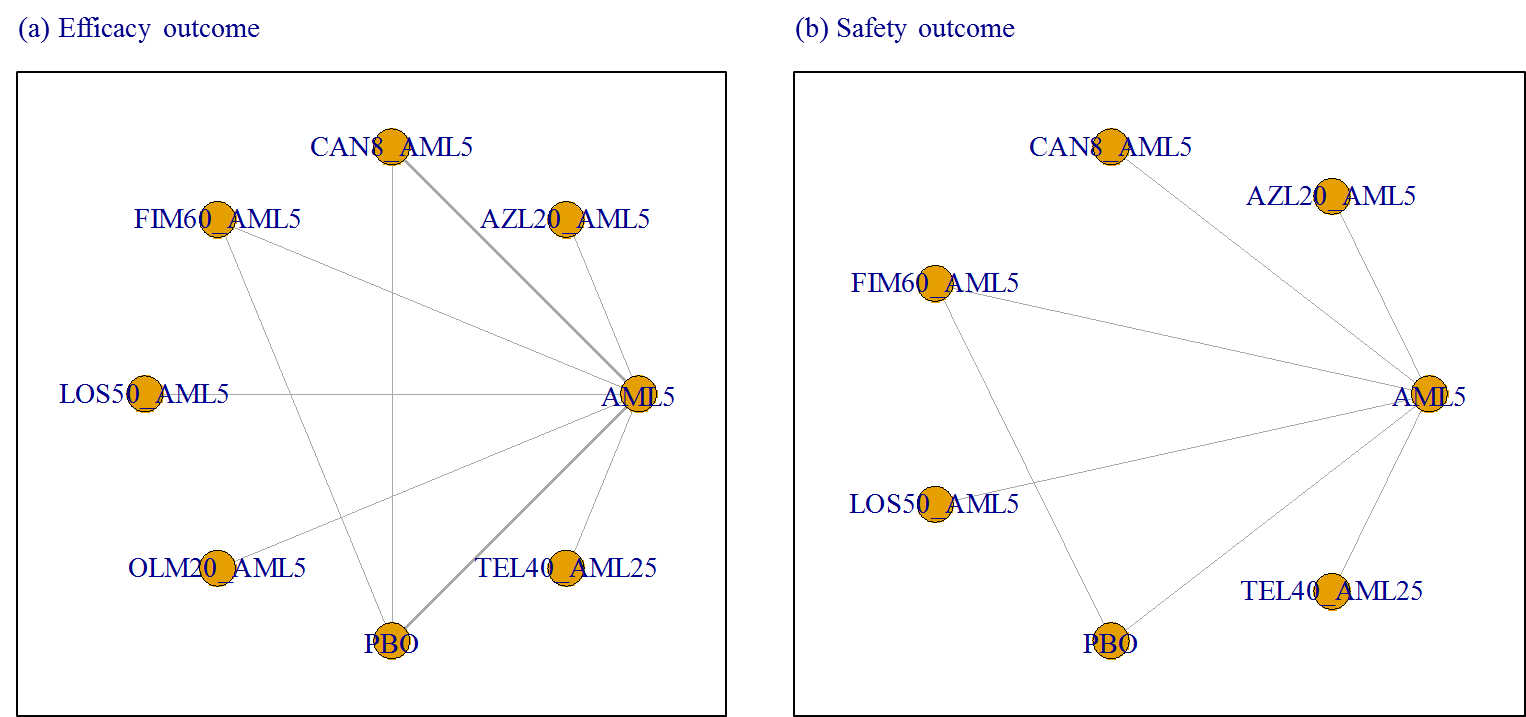


**Appendix 6. SUCRA curves of each comparator for treatment response and TEAE comparison.**

1. **Treatment response**

1. **Treatment emergent adverse event**

**Appendix 7. Direct treatment effect on changes of SBP and DBP from baseline**

1. Systolic blood pressure


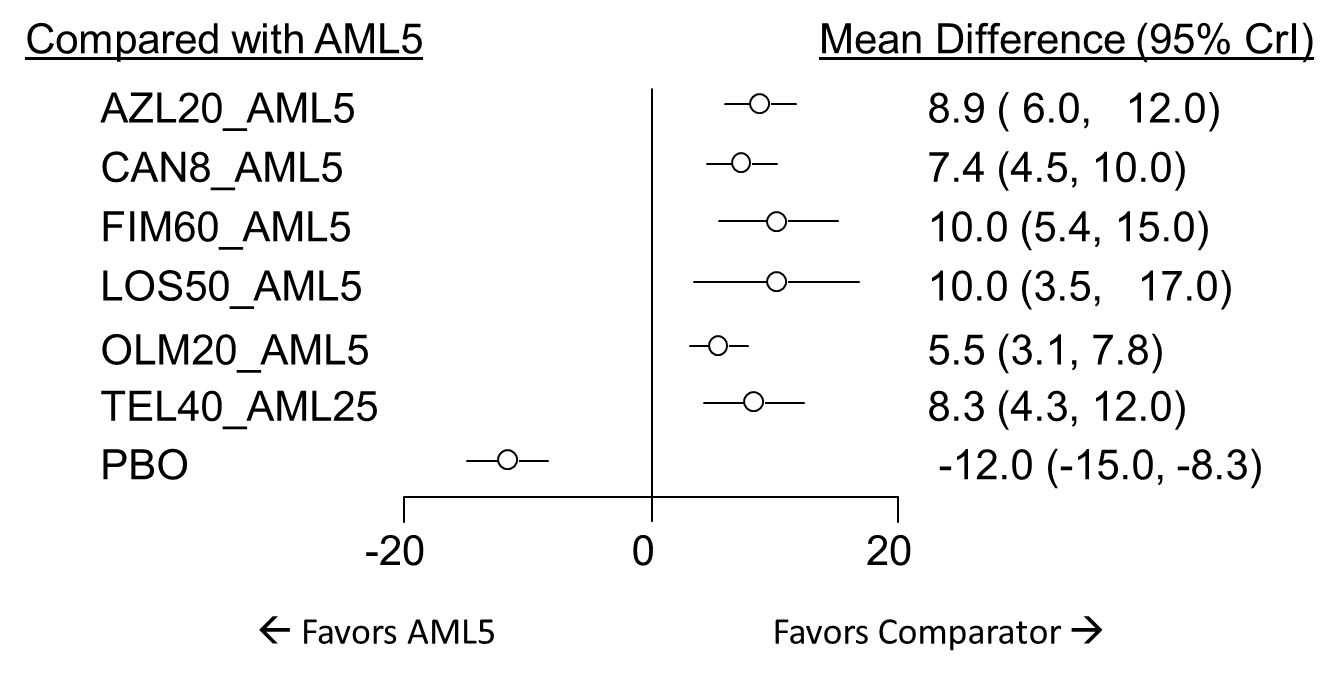


1. Diastolic blood pressure


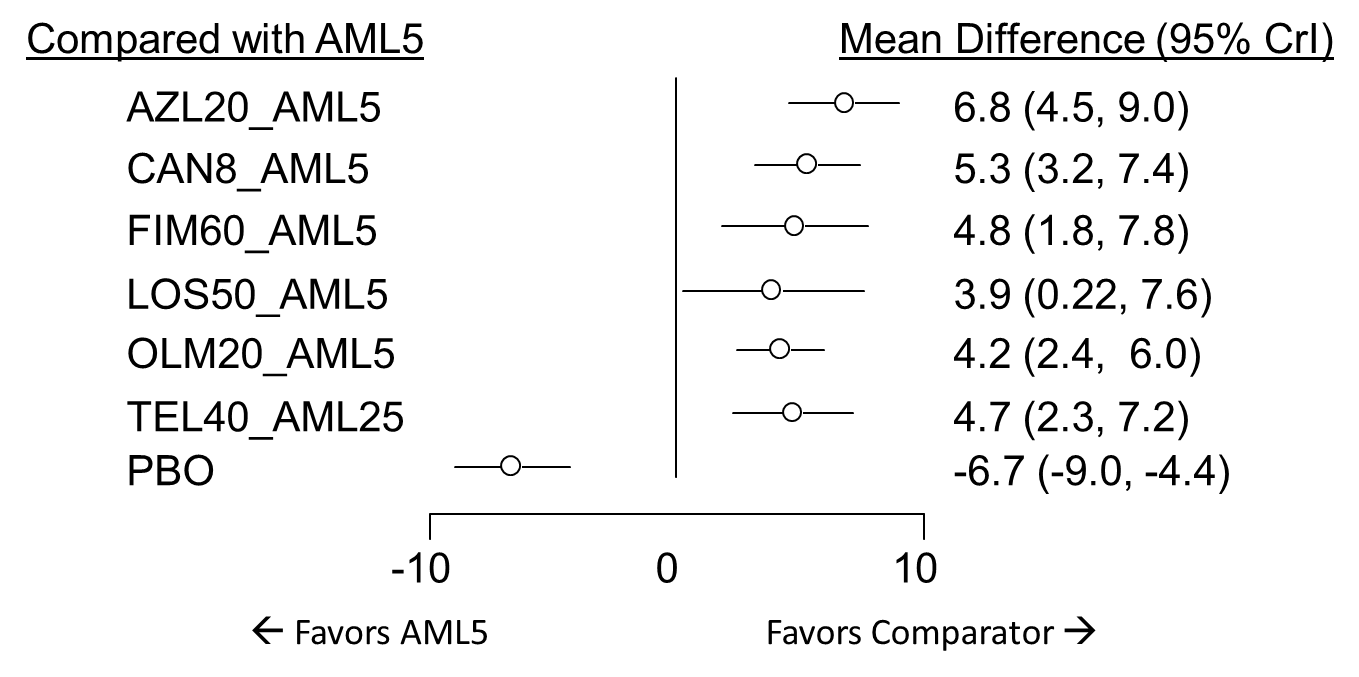


**Appendix 8. Direct and indirect treatment effect on changes of SBP and DBP from baseline**

1. Systolic blood pressure

| **AML5** | 8.89  (5.95, 11.81) | 7.35  (4.5, 10.22) | 10.24  (5.38, 15.11) | 10.23  (3.62, 16.86) | 5.49  (3.14, 7.83) | -11.68  (-15.05, -8.32) | 8.33  (4.28, 12.39) |
| --- | --- | --- | --- | --- | --- | --- | --- |
| **-8.89  (-11.81, -5.95)** | **AZL20_AML5** | -1.55  (-5.62, 2.53) | 1.35  (-4.33, 7.08) | 1.34  (-5.95, 8.57) | -3.41  (-7.13, 0.35) | -20.57  (-25.04, -16.14) | -0.56  (-5.54, 4.47) |
| **-7.35  (-10.22, -4.5)** | 1.55  (-2.53, 5.62) | **CAN8_AML5** | 2.91  (-2.45, 8.27) | 2.88  (-4.33, 10.11) | -1.86  (-5.58, 1.82) | -19.03  (-22.84, -15.23) | 0.98  (-3.95, 5.95) |
| **-10.24  (-15.11, -5.38)** | -1.35  (-7.08, 4.33) | -2.91  (-8.27, 2.45) | **FIM60_AML5** | -0.02  (-8.26, 8.21) | -4.74  (-10.21, 0.64) | -21.94  (-26.61, -17.24) | -1.91  (-8.22, 4.43) |
| **-10.23  (-16.86, -3.62)** | -1.34  (-8.57, 5.95) | -2.88  (-10.11, 4.33) | 0.02  (-8.21, 8.26) | **LOS50_AML5** | -4.73  (-11.81, 2.31) | -21.9  (-29.38, -14.53) | -1.89  (-9.61, 5.91) |
| **-5.49  (-7.83, -3.14)** | 3.41  (-0.35, 7.13) | 1.86  (-1.82, 5.58) | 4.74  (-0.64, 10.21) | 4.73  (-2.31, 11.81) | **OLM20_AML5** | -17.18  (-21.27, -13.05) | 2.84  (-1.84, 7.53) |
| **11.68  (8.32, 15.05)** | 20.57  (16.14, 25.04) | 19.03  (15.23, 22.84) | 21.94  (17.24, 26.61) | 21.9  (14.53, 29.38) | 17.18  (13.05, 21.27) | **PBO** | 20.02  (14.78, 25.29) |
| **-8.33  (-12.39, -4.28)** | 0.56  (-4.47, 5.54) | -0.98  (-5.95, 3.95) | 1.91  (-4.43, 8.22) | 1.89  (-5.91, 9.61) | -2.84  (-7.53, 1.84) | -20.02  (-25.29, -14.78) | **TEL40_AML25** |

1. Diastolic blood pressure

| **AML5** | 6.79  (4.55, 9.03) | 5.32  (3.2, 7.45) | 4.81  (1.83, 7.77) | 3.89  (0.22, 7.57) | 4.2  (2.42, 5.99) | -6.71  (-9.02, -4.37) | 4.74  (2.28, 7.17) |
| --- | --- | --- | --- | --- | --- | --- | --- |
| **-6.79  (-9.03, -4.55)** | **AZL20_AML5** | -1.47  (-4.59, 1.61) | -1.99  (-5.73, 1.76) | -2.91  (-7.18, 1.39) | -2.59  (-5.45, 0.25) | -13.49  (-16.73, -10.23) | -2.06  (-5.4, 1.26) |
| **-5.32  (-7.45, -3.2)** | 1.47  (-1.61, 4.59) | **CAN8_AML5** | -0.52  (-4, 2.97) | -1.42  (-5.66, 2.81) | -1.13  (-3.87, 1.66) | -12.02  (-14.8, -9.25) | -0.58  (-3.82, 2.65) |
| **-4.81  (-7.77, -1.83)** | 1.99  (-1.76, 5.73) | 0.52  (-2.97, 4) | **FIM60_AML5** | -0.91  (-5.64, 3.8) | -0.6  (-4.07, 2.88) | -11.5  (-14.35, -8.65) | -0.07  (-3.92, 3.78) |
| **-3.89  (-7.57, -0.22)** | 2.91  (-1.39, 7.18) | 1.42  (-2.81, 5.66) | 0.91  (-3.8, 5.64) | **LOS50_AML5** | 0.31  (-3.77, 4.39) | -10.59  (-14.95, -6.24) | 0.84  (-3.57, 5.25) |
| **-4.2  (-5.99, -2.42)** | 2.59  (-0.25, 5.45) | 1.13  (-1.66, 3.87) | 0.6  (-2.88, 4.07) | -0.31  (-4.39, 3.77) | **OLM20_AML5** | -10.89  (-13.85, -7.95) | 0.54  (-2.5, 3.57) |
| **6.71  (4.37, 9.02)** | 13.49  (10.23, 16.73) | 12.02  (9.25, 14.8) | 11.5  (8.65, 14.35) | 10.59  (6.24, 14.95) | 10.89  (7.95, 13.85) | **PBO** | 11.43  (8.07, 14.8) |
| **-4.74  (-7.17, -2.28)** | 2.06  (-1.26, 5.4) | 0.58  (-2.65, 3.82) | 0.07  (-3.78, 3.92) | -0.84  (-5.25, 3.57) | -0.54  (-3.57, 2.5) | -11.43  (-14.8, -8.07) | **TEL40_AML25** |
